# 4D Visualization of replication foci in mammalian cells corresponding to individual replicons

**DOI:** 10.1038/ncomms11231

**Published:** 2016-04-07

**Authors:** V. O. Chagin, C. S. Casas-Delucchi, M. Reinhart, L. Schermelleh, Y. Markaki, A. Maiser, J. J. Bolius, A. Bensimon, M. Fillies, P. Domaing, Y. M. Rozanov, H. Leonhardt, M. C. Cardoso

**Affiliations:** 1 Department of Biology, Technische Universitaet Darmstadt, Darmstadt 64287, Germany; 2 Laboratory of chromosome stability, Institute of Cytology, St. Petersburg 194064, Russia; 3 Cancer Research UK London Research Institute, Clare Hall Laboratories, South Mimms EN6 3LD, UK; 4 Micron Advanced Bioimaging Unit, Department of Biochemistry, University of Oxford, OX1 3QU Oxford, UK; 5 Center for Integrated Protein Science at the Department of Biology, Ludwig Maximilians University Munich, Planegg-Martinsried 82152, Germany; 6 Genomic Vision, Bagneux 92220, France; 7 Max Delbrueck Center for Molecular Medicine, Berlin 13125, Germany; 8 Charité—Universitaetsmedizin, 13353 Berlin, Germany

## Abstract

Since the pioneering proposal of the replicon model of DNA replication 50 years ago, the predicted replicons have not been identified and quantified at the cellular level. Here, we combine conventional and super-resolution microscopy of replication sites in live and fixed cells with computational image analysis. We complement these data with genome size measurements, comprehensive analysis of S-phase dynamics and quantification of replication fork speed and replicon size in human and mouse cells. These multidimensional analyses demonstrate that replication foci (RFi) in three-dimensional (3D) preserved somatic mammalian cells can be optically resolved down to single replicons throughout S-phase. This challenges the conventional interpretation of nuclear RFi as replication factories, that is, the complex entities that process multiple clustered replicons. Accordingly, 3D genome organization and duplication can be now followed within the chromatin context at the level of individual replicons.

## Abstract (web summary)

Whether replication happens at individual replicons or in replication factories is a controversial debate. Here the authors use super-resolution microscopy and analysis of replication fork speed to present evidence in favour of replicons.

## Graphical abstract

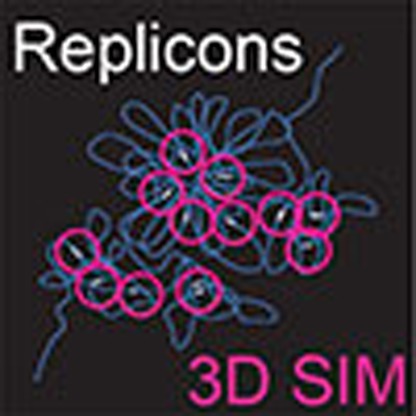

Genomic DNA is duplicated during the S-phase of the eukaryotic cell cycle. At the chromatin fibre level, DNA replication can be characterized by the location on the DNA molecule where the DNA synthetic complexes (replisomes) are assembled and replication is initiated (the so-called origin of replication) and by the actual positions where DNA synthesis occurs at any given moment, termed replication forks^1^. Only a subset of potential origins of replication will be activated in the individual cell in a given cell cycle^2^,^3^,^4^. Each activated origin of replication normally gives rise to two replication forks that drift apart along the template DNA. Initiation of DNA synthesis at a particular origin of replication provides a functional definition of replicon as a chromosome segment replicated as a result of a single initiation event in a particular cell cycle. To duplicate the whole genome in a reasonable time, multiple replicons must operate in parallel at any given time point during S-phase. Data regarding replicon arrangement, size and the rate of replication fork movement, were originally obtained from pattern analysis of tritiated thymidine-labelled tracks of replication forks on extended DNA molecules^5^,^6^,^7^. These DNA autoradiography findings suggested that the genome replicates via clusters of small (50–300 kbp) synchronously activated replicons^8^,^9^. The total number of replicons activated during S-phase was indirectly estimated as 20,000–50,000 (refs 10, 11, 12).

At the cell nucleus level, focal sites of DNA synthesis, hereafter called replication foci (RFi) can be visualized by either labelling replisome components or by detecting sites of nucleotide incorporation upon pulse labelling^13^. The spatial pattern of subnuclear distribution of RFi undergoes dynamic changes during S-phase progression and is characteristic for the different S-phase sub-stages^10^,^13^,^14^. General principles of DNA replication were studied using the analysis of various RFi characteristics, such as their number, brightness, size, lifetime and their intranuclear distribution^10^,^11^,^12^,^13^,^14^,^15^,^16^,^17^,^18^. Up to six distinct patterns of RFi could be distinguished in cycling somatic cells^10^,^19^, although more commonly S-phase was subdivided into early, middle and late stages: Se, Sm and Sl, respectively^20^,^21^,^22^.

Notably, the number of RFi that was observed in each S-phase pattern with conventional microscopic techniques^13^,^23^ was much smaller than the estimated number of active replicons leading to the conclusion that each RF contained multiple replicons^11^,^12^,^13^,^14^,^15^,^16^,^21^,^23^. The stability of RFi over several cell cycles and characteristics of their brightness suggested a relation of nuclear RFi to tandem clusters of synchronously activated replicons described on DNA fibres^12^.

In parallel, the concept of ‘replication factories' arose from electron microscopy observations of localized incorporation of replication label and accumulation of replication proteins in ∼150 nuclear sites^24^, which were similar to the reported numbers of RFi and followed the dynamics of RFi patterns during S-phase^25^. As a result, it was suggested that genome duplication occurred by sliding the template DNA of multiple replicons through composite polymerizing sites of each factory immobilized at the nuclear matrix^25^.

On the basis of these initial studies, RFi were for decades considered as complex functional–structural units of chromatin that contained multiple replicons^26^,^27^.

Studies using fluorescence halo technique revealed a dynamic relationship between replicon size and the size of chromatin loops^28^,^29^,^30^ providing a link between the organization of DNA replication and the structural organization of chromatin. As a result it has been hypothesized that metazoan genome is duplicated by synchronous processing of multiple loops within chromatin domains organized around replication factories^31^.

A comprehensive three-dimensional (3D) analysis of elementary replication units throughout different S-phase stages in mammalian cells was compromised by the limited resolution of optical microscopy. Electron microscopy studies, although less limited in resolution, relied on precarious calculations to estimate the total number of nuclear RFi on the basis of data obtained from partial sections of nuclei^32^,^33^. Accordingly, development of new approaches was essential to close the gap between the data obtained in conventional microscopic and DNA fibre studies on genome replication in higher eukaryotes. Recent advances in super-resolution microscopy provided tools for detailed optical analysis of replication structures in 3D-preserved nuclei^34^,^35^. Although, various high-resolution microscopy techniques led to an increase in the observed numbers of RFi^34^,^36^ 3D-structured illumination microscopy (3D-SIM) proved to be the most suitable approach allowing multicolour 3D detection of replication sites in spatially preserved nuclei^34^. Importantly, the corresponding eightfold increase in 3D resolution posed additional challenges since high throughput analysis and quantification of nuclei containing thousands of RFi was impossible without developing and validating computer-assisted automated approaches.

To re-evaluate the above replication factory concept and test the hypothesis that replicons and not replicon clusters may in fact represent the *in situ* elementary units of DNA replication; in this study, we perform a comprehensive super-resolution analysis of RFi in somatic human and mouse cells. RFi are visualized both by labelling newly synthesized DNA and PCNA as a crucial replisome component. We complement the RFi analysis with quantifications of genome size, S-phase duration and measurements of molecular replicon characteristics of the same cells to overcome inaccuracy through indirect estimates. Using newly developed protocols for robust RFi quantification, we demonstrate that comparable numbers of several thousands of RFi are active throughout all S-phase stages. The combined consideration of the experimental data show that conventionally observed RFi can be optically resolved down to single replicons in all S-phase sub-stages. Our findings imply that S-phase dynamics is primarily dictated by chromatin folding and individual synthetic complexes independently ‘read' and ‘copy' the underlying chromatin units^37^.

## Results

### Kinetic analysis of cell cycle characteristics

**Figure 1:**
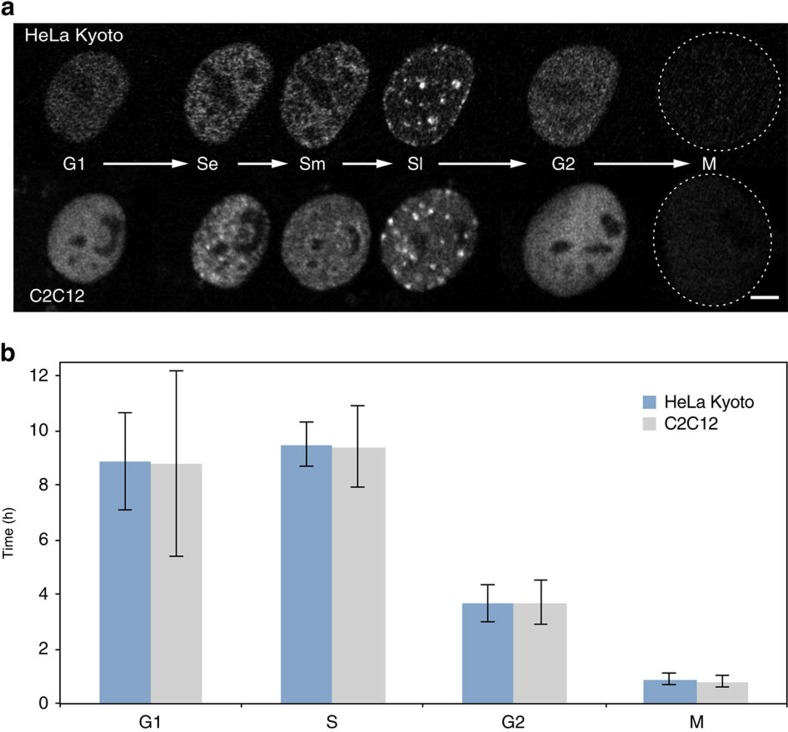
Direct measurement of cell cycle kinetics. (**a**) Patterns of PCNA distribution in all cell cycle stages imaged with live-cell time-lapse microscopy of human HeLa Kyoto (top) and mouse C2C12 cells (bottom). S-phase stages are further subdivided into early (Se) mid (Sm) and late (Sl). (**b**) Duration of the cell cycle phases (mean±s.d.; additional data in Table 1) measured from time-lapse microscopy analysis as shown in **a** (see also Supplementary Movie 1). Error bars represent s.d., number of replicates for human cells G1: 31, S: 30, G2: 27, M: 26; mouse cell replicates, G1: 20, S: 16, G2: 5, M: 10. Scale bar, 5 μm.

To overcome the inaccuracy that arises from indirect estimates we performed direct live-cell analysis of the cell cycle parameters for the newly generated human cell lines (Supplementary Fig. 1 and Supplementary Note 1), as well as for the previously characterized mouse cell line^21^. To measure the duration of all cell cycle stages, we obtained time-lapse series of confocal images from live cells every 15–20 min for at least one complete cell cycle. The absence of phototoxicity-derived effects was supported by two lines of evidence: first, cells commonly entered into mitosis after being illuminated for the whole-cell cycle (Fig. 1a and Supplementary Movie 1); and second, the cell cycle duration (22.6 h) measured from microscopic images of live cells and the time needed for the culture to double in the absence of illumination were essentially the same (Fig. 1b and Table 1). The different cell cycle stages were classified on the basis of sequential appearance of characteristic PCNA distributions (Fig. 1a). Cells with uniformly distributed nuclear PCNA foci were classified as being in early S-phase (Se), perinucleolar foci rings were used as main marker of mid S-phase cells (Sm) and bright RFi clusters were used to distinguish cells in late S-phase. The onset of mitosis was manifested by the dilution of PCNA signal and changes in the shape of the cell that were also evident in phase contrast images. (Fig. 1a and Supplementary Movie 1). We used the information on the preceding or the following cell cycle stage to classify nuclei with homogeneous PCNA distribution as G1 or G2 stage.

Despite differences in their karyotypes, the human and mouse cells had comparable cell cycle and S-phase (9.5 h) durations (Fig. 1b and Table 1).

### Genome size measurements

**Figure 2:**
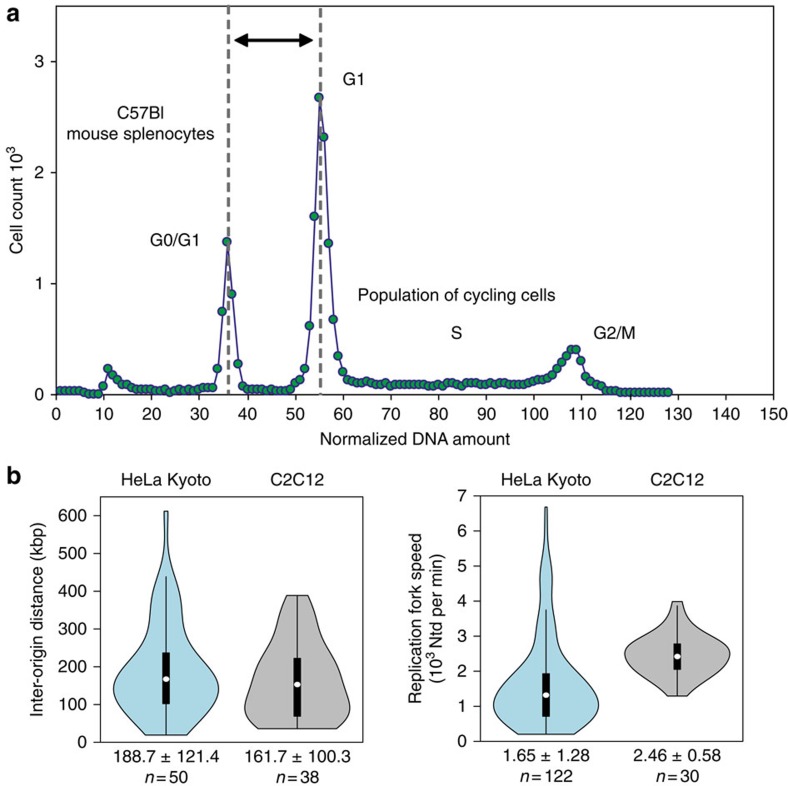
Genomic DNA content and DNA fibre analysis of replicons. (**a**) DNA flow cytometry histogram of ethidium bromide/olivomycin stained HeLa Kyoto mCherry-PCNA expressing cells admixed with C57Bl mouse splenocytes is shown. The peak at channels 34–38 corresponds to the G1/G0 peak of non-cycling splenocytes. HeLa Kyoto cell cycle distribution is represented by a typical DNA flow cytometry histogram consisting of G1, S-phase and G2/M populations. To calculate the amount of genomic DNA in the cycling cell line, G1/G0 peak of mouse splenocytes and G1 peak of the cell line were approximated with Gaussian distributions and the relative position of the G1 peak was calculated (for details see methods and Table 2). (**b**) Cells were pulse labelled with IdU for 30 min, followed by a 30 min CldU pulse. Whole-genome DNA was extracted under gentle conditions and single DNA fibres were stretched with the constant factor of 2 kbp per μm. Incorporated nucleotides were immunostained and signals acquired in a wide-field microscope. Fluorescent tracks were measured by hand and used to calculate mean IOD and RFS. For details see methods; Supplementary Fig. 2 and Tables 3 and 4.

A cell line represents a lineage of cells capable of unlimited proliferation cycles in culture. Transformation of these cells can often lead to changes in chromosome numbers and/or genome size. Accordingly, it was necessary to determine the amount of genomic DNA for each cell line using normal diploid mouse cells as a reference (Fig. 2a). All cell lines had non-diploid karyotypes. The HeLa Kyoto cell lines expressing GFP-PCNA or mCherry-PCNA had very close genome sizes of 9.7 and 9.8 Gbp, respectively (Fig. 2a and Table 2). In view of their common origin and close similarity, these cell lines were used interchangeably in further experiments. C2C12 cells expressing GFP-PCNA were quasi-tetraploid^38^, with 11.4 Gbp genome size (Fig. 2a and Table 2).

### Quantification of molecular characteristics of replicons

The time needed to duplicate a genome is primarily determined by: (i) the average spacing of active replication origins; and (ii) the rate of DNA synthesis. To measure both, inter-origin distances (IODs) and the rate of chain elongation (replication fork speed, RFS), we performed labelling of the cells by consecutive incubation with two thymidine analogues—IdU and CldU (Supplementary Fig. 2) and took advantage of the DNA combing procedure that led to uniform stretching of DNA fibres^39^. This procedure produces on average stretched DNA fibres of 250–500 kbp in length^39^, with a fibre length of at least ∼200 kbp essential for relevant RFS and IOD experimental estimates^40^. The average IOD was comparable in mouse and human cells (189 and 162 kbp for HeLa Kyoto and C2C12, respectively; Fig. 2b and Tables 3 and 4). On the other hand, the average RFS was very different with 1.65 kbp min^−1^ in human cells and 2.46 kbp min^−1^ in mouse cells (Fig. 2b and Tables 3 and 4). The consistency and statistical relevance of the sample size was verified by a sliding average test (Supplementary Fig. 2C and Supplementary Note 2).

### Visualization of DNA replication sites in cells using 3D-SIM

**Figure 3:**
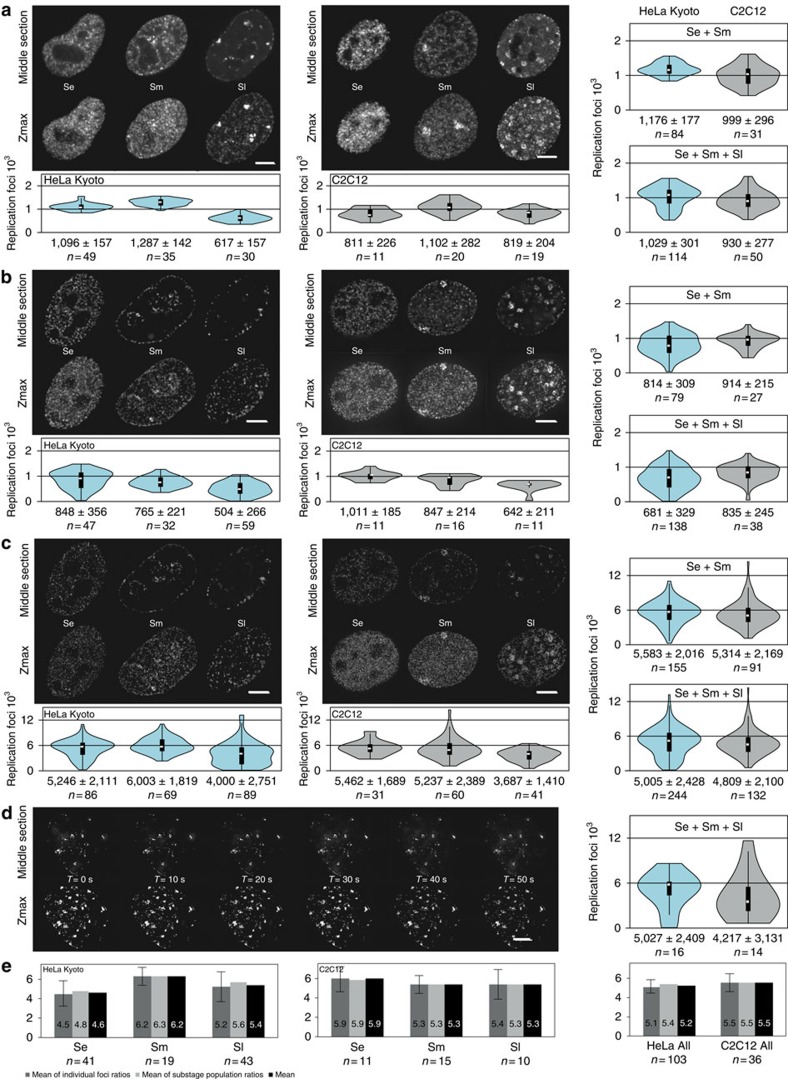
3D quantification of RFi numbers throughout S-phase. (**a**) Mid sections and maximum intensity z-projections (Zmax) of spinning disk confocal microscopy images of human (HeLa Kyoto) and mouse (C2C12) cells as indicated representative of the three major S-phase patterns—early (Se), mid (Sm) and late (Sl)—are shown. Scale bar, 5 μm. Numbers (mean±s.d.; summary of the data in Supplementary Fig. 5 and Supplementary Table 1) of nuclear RFi quantified as described in Supplementary Fig. 3 are plotted separately for each of the three major S-phase patterns as well as the pooled data for Se and Sm and the whole S-phase. N indicates the number of cells analysed. (**b**) As in **a** representative images from wide-field deconvolution microscopy and corresponding RFi numbers. Scale bar, 5 μm. (**c**) As in **a** representative images from 3D-SIM and corresponding RFi numbers. Scale bar, 5 μm. (**d**) Time series (see also Supplementary Movie 2) of live mouse cells imaged using 3D-SIM and corresponding RFi numbers for mouse and also for human cells. Scale bar, 5 μm. (**e**) Histogram of RFi ratios from super-resolution versus conventional microscopy. Ratios were calculated either per individual cell (dark grey) or from all cells pooled (light grey). In addition, both data sets were combined (black). Given error bars represent the s.d.

Next, we visualized the replication sites *in situ* throughout S-phase in both human and mouse cells at different light-microscopy resolution levels. Samples for 3D-SIM super-resolution imaging with RFi labelled by nucleotide incorporation were prepared by growing proliferating cultures of mouse and human cells in the presence of different cell permeable thymidine analogues (EdU or BrdU). In addition, RFi labelled by fluorescent PCNA to highlight replisomes were imaged in the same cells. The moderate expression levels of fluorescent PCNA in the stable cell lines essential to ensure unaltered cell cycle dynamics, were not strong enough to utilize the full potential of the 3D-SIM method^34^,^35^,^41^. Hence, an additional staining with anti-PCNA antibodies was performed to enhance the PCNA signal. To cover all optical resolution levels, we also acquired laser scanning confocal microscopy images and generated conventional wide-field epifluorescence images from the raw data sets obtained at the 3D-SIM system as well as the respective deconvolved images. In most cases, not only fixed and stained cell images but also live-cell images were acquired, as shown in Fig. 3. All characteristic S-phase patterns described in conventional wide-field and confocal microscopy could be identified in super-resolution images.

### Pan S-phase quantification of replication foci numbers

Using newly developed computational approaches for RFi quantification (Supplementary Note 3, Supplementary Fig. 3 and ref. 42), we next counted the numbers of RFi (nucleotide and protein labelling) for every major S-phase stage at the different optical resolution levels (Fig. 3 and Supplementary Table 1).

Application of the counting protocol^42^ to Z-stacks of confocal images led to the identification of on average 1,096 and 811 RFi per human and mouse cell in early S-phase comparable to previous reports. Mid S-phase cells yielded moderately higher RFi numbers, whereas this number decreased in late S-phase when the characteristic pattern of bigger and brighter RFi appeared (Fig. 3a). Similarly to confocal data, the number of RFi in deconvolved wide-field image stacks of early S-phase cells was 848 and 1,011 for human and mouse cells, respectively (Fig. 3b). Some S-phase stage fluctuation in RFi numbers (from 4,000 to 6,003 and 3,687 to 5,462 for human and mouse cells, respectively) could be found in 3D-SIM image stacks with mid S-phase numbers higher in human cells and early S-phase numbers higher in mouse cells (Fig. 3c). This suggests cell type or species-specific differences in S-phase dynamics and stresses the importance of complementing the *in situ* RFi measurements with a thorough characterization of genome size, IOD and RFS in the same cells. In both, human and mouse cells, RFi numbers declined toward late S-phase, due to prominent clustering of a substantial portion of RFi compromising proper separation and identification of individual RFi (Supplementary Fig. 4 and Supplementary Note 4) as well as decaying number of replicons towards the end of S-phase. To obtain an estimate of maximum number of RFi, we therefore excluded late S-phase cells from further calculations and averaged RFi numbers observed in early and mid segments of S-phase. With PCNA (replisome) labelling we detected on average slightly higher numbers of RFi for both mouse and human cell lines as compared with nucleotide labelling (Supplementary Fig. 5 and Supplementary Note 4). In addition, we acquired 3D-SIM time-lapse images of RFi in live cells labelled with GFP-PCNA (Fig. 3d). The RFi counted from the live super-resolution analysis yielded numbers close to the fixed-cell analysis albeit, in view of the rapid signal degradation as a consequence of GFP photobleaching, generally lower. We further observed inherent variability in RFi numbers between individual cells. Such variability may be a unique feature uncovered by high-resolution imaging of replication structures, which previously, at lower resolution, was manifested as variability in intensity of RFi^12^,^21^,^43^. Independently from the variability in individual replicon characteristics and in RFi numbers per cell, genome duplication must be completed within a normal S-phase length of 9.5 h.

As the 3D-SIM system, in addition to reconstructed super-resolved image stacks, allows to simultaneously generate the corresponding wide-field (and optionally deconvolved) image stacks, we were able to directly compare the total per cell RFi from different imaging-resolution conditions of the very same set of cells. We calculated both the ratios of RFi within every single cell or pooled the data from many cells together and calculated the population RFi ratio. Both ratios (Fig. 3e) perfectly agreed and indicated that on average a RFi detected at conventional light-microscopy resolution corresponds to 5.2 and 5.5 (nano)RFi at super-resolution imaging for human and mouse respectively. Moreover, these numbers varied between 4.5 and 6.3, with the higher values in mid S-phase of human cells.

### Genome duplication parameters reveals single replicons

**Figure 4:**
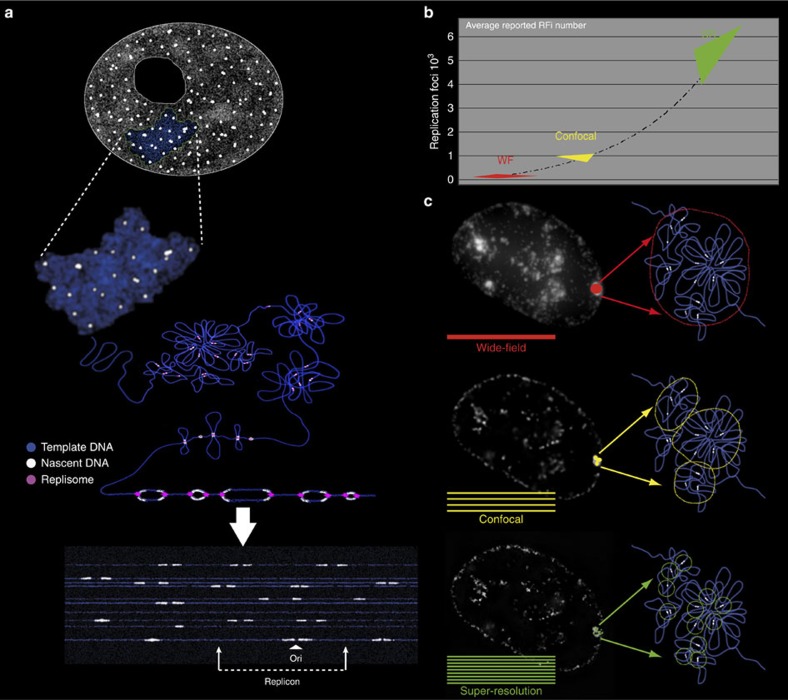
Replication sites dissected by super-resolution microscopy in the mammalian nucleus correspond to individual replicons. (**a**) A cartoon showing how replication sites/units can be seen at different levels of chromatin compaction from the extended DNA fibres to the 3D-preserved whole-cell (nucleus) level. (**b**) Increase in RFi numbers driven by resolution improvements in microscopy during the past three decades^42^. WF: wide field; SR: super-resolution microscopy. (**c**) Microscopic images and corresponding cartoon interpretation of replication sites in the mammalian nucleus imaged at different levels of resolution. For summary of experimental numbers and calculations see Table 5.

Finally, we integrated the numbers of all experimentally determined parameters for mouse and human cells to evaluate the relation of 3D-SIM-resolved RFi to elementary replication units (Fig. 4 and Table 5).

We used unsynchronized cells to measure distance between adjacent origins (IOD) activated at different moments of S-phase. The total number of replicons (equivalent to origins that become active) during S-phase equals to the genome size divided by the average IOD (used as an approximation for replicon size). This results on 51,404 and 70,501 replicons needed in total to duplicate the human and mouse cell genome, respectively, which is compatible with reported estimates^10^,^11^,^12^. The subset of simultaneously active replicons at any given time is proposed to be determined by the number of available limiting factor molecules^3^,^4^. The total duration of DNA synthesis of an average bidirectional replicon (replicon ‘lifetime') was calculated by dividing IOD by two times the RFS, resulting in ∼57 min in human cells and ∼33 min in mouse cells, which corresponds to the period of time each limiting factor is occupied. The number of times each limiting factor molecule is reused can be estimated as the duration of S-phase divided by the average replicon lifetime. The latter results in 10 and 17 cycles for each limiting factor molecule during the complete S-phase in HeLa and C2C12 cells, respectively.

Since IOD measurements can be more affected by DNA fibre length than RFS measurements^40^, we used primarily RFS data to estimate the number of replicons needed to replicate the whole genome. The number of replication forks that need to operate in parallel during S-phase can be calculated by dividing the time needed to duplicate the whole genome by a single replication fork (genome size divided by the average RFS) by the measured S-phase duration. This calculation showed that ∼10,000 (human cells) and 8,000 (mouse cells) forks or half as many bidirectional replicons (∼5,000 and ∼4,000) operated in parallel in human and mouse cells, respectively (Table 5). The numbers of RFi counted by super-resolution microscopy (5,583 and 5,314 for human and mouse cells, respectively) can now be directly compared with the predicted numbers of simultaneously active replicons (5,149 and 4,108 for human and mouse cells, respectively). The outcome is a quotient of calculated simultaneously active replicons to the measured average number of RFi in 3D-SIM images for both mouse and human cells (Table 5). The robustness of our calculation is verified by a calculated mean squared error (MSE, Table 5) using a simplified version of the Gaussian error formula (see, equation 3 in the Materials and methods). The quotient in human cells of 0.92 is accompanied by a MSE of 0.2 and the quotient in mouse cells of 0.77 has a MSE of 0.3 respectively. Those MSE take into account the variances of the measured genome size (Table 2), the measured fork speed (Table 3) and the measured S-phase length (Table 1). The difference of the average number of replicons per single RF between human (0.92) and mouse (0.77) cells, likely arise from differential clustering of replication forks in particular cell types and/or S-phase sub-periods when some RFi can contain individual replication forks.

All in all, we conclude that, for all S-phase patterns, the majority of nuclear replication sites were resolved down to the level of single replicons with a portion of spatially separated single replication forks.

## Discussion

In this study, we present a comprehensive examination of DNA replication in mammalian cells including various resolution levels of optical microscopy. Special effort was made to control for all inaccuracies that could affect the outcome of the analysis and characterization of RFi in super-resolution images.

First, we took advantage of mammalian cell lines stably expressing fluorescent replication factors and performed confocal live-cell microscopy to directly characterize the temporal S-phase dynamics in these cells. We further measured genome size for each cell line used in our experiments and analysed the molecular characteristics of replicons in the same cells. To quantify RFi numbers in super-resolution images, we developed and verified user-independent protocols for 3D RFi segmentation and counting. We compared RFi quantifications results with respect with the other parameters measured for identical cells.

In both cell lines we detected on average five thousands RFi at any S-phase sub-stage using super-resolution imaging (Fig. 3). Combining all the experimental data together, we concluded that the majority of RFi represent single replicons at 3D-SIM resolution with a number of optically resolved single replication forks.

According to the limiting factor concept, the number of active replicons at any given S-phase time-point is determined by the pool of available limiting factors. If for these replicons it is assumed that the corresponding origins of replication are activated during a short-time window of S-phase, the time of synthesis of the whole subset will roughly correspond to the lifetime of an average replicon, which we estimated as 57 min for human and 33 min for mouse cells. S-phase progression can be modelled as sequential activation of subsets of origins and DNA synthesis in the corresponding pluralities of replicons. It is unlikely, however, that there are distinct classes of origins, which are initiated strictly one after the other. A more realistic scenario is that origin firing of adjacent replicons in the next subset starts before replicons from the previous subset complete DNA synthesis, leading to replicons from multiple subsets being active in parallel^2^,^44^. Both sequential synchronous and asynchronous modes of origin firing would nonetheless lead to identical average numbers of simultaneously active replicons (Supplementary Fig. 6). Similarly, the subdivision of S-phase into three major discrete sub-stages (Se, Sm and Sl) is an oversimplification of real RFi dynamics, which very likely represents a continuous spreading of replication onto non-replicated chromosome segments and corresponding gradual changes in RFi patterns. DNA flow cytometry histograms (Supplementary Fig. 6 as well as accompanying study^37^) demonstrate the absence of substantial differences in cumulative DNA synthesis intensity throughout S-phase. Therefore, the average estimates of RFi numbers used in our calculations represented a reasonable simplification.

The empirical differences between Se, Sm and Sl RFi numbers may illustrate variations in degree of clustering of replication forks during individual S-phase stages. For example, the portion of RFi containing single-replication forks may be higher than average during early S-phase in mouse C2C12 and during middle S-phase in HeLa Kyoto cells. In the latter cases, 3D-SIM may still not completely resolve all RFi leading to their underestimation (Fig. 3c and Supplementary Fig. 4). We also observed cell-to-cell variability in RFi numbers and S-phase duration, as well as average spacing of origins and RFS^45^. Differences in RFi numbers in Se, Sm and Sl may also be associated with corresponding changes in RFS and IOD. Nonetheless, the mouse myoblasts having a larger genome size but the same S-phase duration (time to duplicate the whole genome) did not compensate by increasing the average number of simultaneously active replicons but rather mainly by tweaking up the DNA synthesis speed. The latter has interesting metabolic implications regarding nucleotide pool availability.

While the intra-S-phase variations of all these parameters are worthy of a separate study, such a detailed analysis will not affect our main conclusions since, despite the reported variability of the above parameters, genome duplication is completed by the end of S-phase.

The observation of spatially separated replication machineries corresponding to individual replicons and replication forks in (live) 3D-preserved cells contradicts the model of S-phase progression based on replication factories as common synthetic centres that process multiple tandem replicons. The term ‘replication factories' was initially coined based on the combined consideration of: (i) small number of RFi, which were nuclease resistant and contained nascent DNA and replication proteins; and (ii) DNA fibre data on organization of replicons in clusters; which taken together suggested that each replication focus ‘was a ‘factory' containing many polymerizing machines' that synchronously processes aggregates of multiple tandem replicons^24^,^26^. The reported clustering of multiple tandem replicons may be a consequence of the inhibitor treatments used in the original DNA fibre and autoradiography studies leading to dormant origin activation^7^,^12^,^27^. Our data show that individual replicons or even single forks can be optically resolved. Therefore, these data suggest that the basics of the replication factories concept are not supported by the improved resolution of imaging and RFi can no longer be considered as complex entities, that is, factories. Accordingly, our data suggest that, at the nuclear level, the process of DNA replication is unlikely to involve assembly of multiple origins of replication at specific aggregate synthesis centres, but the replication machinery rather reads structural aspects of chromatin organization. Chromatin separation into individual replication units may correlate with its organization into topologically associated stable domains^12^,^46^ however not much is known of what determines chromosome organization into TADs.

The idea that chromatin organization can dictate the spatial organization of DNA replication is supported by the data on *de-novo* assembly of new replisomes by a domino effect-like mechanism *in cis*^21^,^44^,^47^. In this scenario, further elaborated in the accompanying study^37^, the sites of assembly, the pattern and dynamics of nuclear RFi will be dictated by the intranuclear folding of the chromatin fibre itself. Accordingly, in physiological conditions (that is, in the absence of replicative stress) replication-related reorganization of chromatin will be limited to local changes of chromatin condensation state, which will be more prominent in compacted heterochromatin. The above model of S-phase progression is also compatible with the reported influence of DNA replication fork movement on the chromatin loop size organization and origin choice in the following cell cycle^29^.

Resolving conventional nuclear RFi down to sites containing single replicons or replication forks implies a modified interpretation of the RFi characteristics that are traditionally analysed in studies of spatio-temporal organization of DNA replication. In this respect, the original meaning of the term ‘replication factory' as a macromolecular complex performing simultaneous synthesis of multiple replicons, needs to be reduced to smaller replisome complexes or even single replisomes, which are assembled on DNA spatially organized within the nucleus.

An inherent component of the replication factory model were clusters of 30 nm chromatin loops arranged at each factory^26^, which were assumed to form rosette-like chromatin subcompartments. Analysis of chromatin interactions using 3C-based technologies^48^ has suggested that, above 11 nm nucleosomal string, there can exist not only canonical 30 nm fibre^49^ but also various higher-level compaction states of interphase chromatin^50^,^51^. Our data and the ensuing model^37^ are compatible with the view that interphase chromatin fibres are organized by complex and dynamic topological looping interactions^52^, which provide a structural framework for DNA metabolism. Based on our comparison of numbers of RFi from conventional and super-resolution microscopy, an average of five replicons correspond to one conventional replication focus (Fig. 3e). This analysis suggests a spatial association of replicons within one Mbp chromatin segment, which likely reflects the spatial chromatin organization of the segment. Nonetheless, genetic continuity would not be mandatory for such an association. As proposed in our accompanying study^37^, the induced domino-like replication origin activation, would implicitly lead to the temporal grouping of active replicons within a chromatin fibre. Further experimental analyses of dynamic relationships between neighbouring RFi will be needed.

Finally, the results presented in this study also suggest that 3D-SIM microscopy is a first-choice approach for multicolour 3D analysis of elementary replication units in eukaryotic cells. Based on 3D-SIM microscopy and multicolour 3D analysis, further experiments need to be designed to address the 3D arrangement of replicons in relation to epigenetic chromatin signatures and other aspects of functional chromatin organization. Our findings and ongoing development of higher spatio-temporal resolution 3D-SIM live systems^53^,^54^,^55^ create a basis for *in vivo* genome duplication analysis in 3D at a single-replicon resolution. Importantly, we present evidence that individual replicons within the chromatin context and not replicon clusters represent the main players of DNA replication. We propose that beyond the 150–200 bp nucleosomal DNA unit, a subsequent order of functional chromatin organization is constituted by the a thousand times larger (150–200 kbp) genome unit functioning as individual replicons during S-phase. Fifty years after the introduction of the replicon concept^56^ individual replicons are again in focus backed by our vastly improved knowledge of chromatin structure and function.

## Methods

### Cell culture

HeLa Kyoto cells^57^ (a kind gift from Jan Ellenberg) were grown in DMEM medium supplemented with 10% FCS, L-glutamine and antibiotics at 37 °C in a humidified atmosphere of 5% CO_2_. Mouse C2C12 myoblasts expressing fluorescently tagged PCNA^21^ were grown in DMEM medium supplemented with 20% FCS, L-glutamine and antibiotics at 37 °C in a humidified atmosphere of 5% CO_2_.

### Generation of cell lines stably expressing fluorescent PCNA

HeLa Kyoto cell lines expressing fluorescent PCNA variants were obtained using the Flp-In system (Invitrogen) based on the Flp site-specific recombinase. Briefly, cells were first transfected with a plasmid bearing a FRT site and the Zeocin resistance gene fused to the *LacZ* gene (pFRT-lacZeo) using PEI transfection^58^. Cells where the plasmid integrated into a chromosome were selected throughout a week on the basis of the newly acquired Zeocin resistance (75 μg ml^−1^) and eight clones with integrated FRT sites were isolated. Beta-galactosidase activity of HeLa Kyoto LacZ stable clones was then verified using X-gal and ONPG (*o*-nitrophenyl-β-D-galactosidase) assays.

HeLa Kyoto FRTLacZ clones with low and high β-galactosidase activity were selected for further transfection with pFRT-B-GPCNA (encoding GFP-PCNA) and pFRT-B-CPCNA (encoding mCherry-PCNA) plasmids and cotransfected with pOG44 Flp-recombinase using Transfectin (BioRad) (Supplementary Fig. 1A). Four hours after transfection the cell culture medium was exchanged and cells were grown for 48 h and selected with 2.5 μg ml^−1^ Blasticidin (Invitrogen).

### Characterization of cell lines expressing fluorescent PCNA

Absence of cell cycle effects was verified by propidium iodide (PI) staining and flow cytometry analysis (Supplementary Fig. 1C). For cell cycle analysis with PI staining, cells were trypsinized, washed with PBS, pelleted and fixed with ice-cold methanol (1–4 h incubated at 4 °C). After fixation, cells were pelleted and resuspended in PBS then treated with RNAseA (Sigma, working concentration: 50 μg ml^−1^) and incubated with PI solution (final concentration 50 μg ml^−1^, 30 min at 4 °C). Samples were run on a BD FACSVantage flow cytometer and the data were analysed using FlowJo software (Tree Star Inc.).

Expression and characteristic S-phase distributions of fluorescent PCNA were verified visually. Colocalization of GFP-PCNA and mCherry-PCNA with active sites of active nuclear replication was confirmed using BrdU labelling and detection that was performed as follows: BrdU (BD Biosciences) was added to the cell culture medium to the final concentration of 100 μM for 30 min, the cells were then washed with PBS and then fixed with 3.7% formaldehyde for 10 min at room temperature; DNA was denatured by DNAseI treatment, anti-BrdU primary mouse antibody (1:5, BD Biosciences, catalog # 347580) and donkey anti-mouse IgG Texas Red (1:200, Jackson Immuno Research Laboratories, catalog # 715-075-151) or goat anti-mouse IgG Alexa Fluor 488 (1:400, ThermoFisher Scientific, catalog # 11001) secondary antibodies; and nuclear DNA was stained with DAPI (0.5 μg ml^−1^), 5 min at room temperature.

Counting of BrdU positive S-phase cells versus non-S-phase cells showed that 34.9% of cells were in S-phase. This number was comparable to the flow cytometry estimates.

For immunoblot analysis (Supplementary Fig. 1B) of the ectopic fusion proteins and the relative amount of the endogenous PCNA, whole-cell lysates were analysed by SDS–polyacrylamide gel electrophoresis, transferred to nitrocellulose membranes and incubated with rat anti-PCNA monoclonal antibodies 16D10 (ref. 59) followed by donkey anti-rat IgG Cy5 (1:200, Jackson Immuno Research Laboratories, catalog # 712-175-153) and detection using a fluorescence scanning imaging system (STORM, GE Healthcare).

### Genome size measurements

To measure the amount of genomic DNA the cells were washed twice with PBS/EDTA buffer, trypsinized and resuspended in Versene solution (0.2 g l^−1^ EDTA(Na4) in PBS). Before staining, cells were counted and mixed with a comparable number of male C57Bl mice splenocytes. For DNA staining, the cellular suspension was supplemented with Triton X-100 (Sigma) to the final concentration of 0.1%, ethidium bromide (Calbiochem) to the final concentration 20 mg ml^−1^ and olivomycin A (MZM) to the final concentration 40 mg ml^−1^ and MgCl_2_ to the final concentration 15 mM, and incubated for 24 h at 4 °C. Measurements were performed using a self-built high-resolution cytometer setup based on a fluorescence microscope and laminar flow chamber^60^. At least three DNA histograms were obtained for each probe. For C57Bl mouse splenocytes used as a standard object, the variation coefficient of DNA histograms was <2.0%. To calculate the average DNA content in a cell population, positions of the peak in the histogram corresponding to the mouse splenocytes and G1 peak of the cell population were determined (Fig. 2a). The error of measurement of the G1 peak position was ≤0.2%. The relative amount of the genomic DNA in each cell line was corrected for human/mouse genome size and female/male differences (factors of 1.06 and 1.016, respectively). The size of genomic DNA in base pairs was calculated based on the estimated amount of DNA in a diploid human genome—7 pg (ref. 61)—with the following formula:


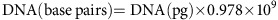


### DNA fibre experiments

Replication labelling and preparation of DNA fibres: Cells were pulse labelled with 100 μM IdU for 30 min, washed two times with PBS, followed by a 30 min 100 μM CldU pulse. Cells were trypsinized, pelleted and resuspended in low-temperature melting agarose to form plugs of 200,000 cells each. Plugs were incubated over night at 50 °C in 0.25 mg ml^−1^ proteinase K in 10% sarcosyl/EDTA, washed in Tris-EDTA buffer twice for 30 min at room temperature. Agarose was digested at 42 °C by two units of β-agarase per plug. Fibres were combed using the Genomic Vision combing machine as follows: in short, a silanized coverslip was incubated in the sample for 5 min. The coverslip was removed at a constant speed of 300 μm s^−1^ with a resulting average fibre length between 250–500 kbp.

*Staining*: DNA fibres were dehydrated in a series of ethanol with increasing concentration and denatured in a 0.5 M NaOH/1 M NaCl solution. After washing with 0.05 M Tris/1 M NaCl and PBS, the incorporated nucleotides were detected with two to four layers of antibodies in 4% BSA/PBS for each 1 h at 37 °C. Primary antibodies: mouse anti-BrdU (1:5, BD Biosciences, catalog # 347580); rat anti-BrdU (1:25, Harlan Sera-Lab, catalog # OBT0030). Secondary antibodies: goat anti-mouse IgG Alexa 488 (1:200, ThermoFisher Scientific, catalog # 11001); and donkey anti-rat IgG Cy3 (1:200, Jackson Immuno Research Laboratories, catalog # 712-165-153). Third antibody: horse anti-goat IgG biotin (1:200, Vector Laboratories, catalog # BA-9500). Fourth layer: Streptavidin-Alexa 488 (1:200, Invitrogen, catalog # S11223). Stained DNA fibres were mounted in Vectashield (Invitrogen).

*Microscopy*: Epifluorescence images were obtained using an Axiovert 200 microscope (Zeiss) with a × 40/1.4 NA Plan-Apochromat oil immersion objective lens (Zeiss) and a cooled 12-bit charge-coupled device camera (Sensicam).

*Image analysis*: The brightness and colour of each image was adjusted with ImageJ^62^. It should be considered that the several pictures of one fibre look equal in brightness and contrast. IdU was set to green, CldU to red. Alignment of the images of the same fibre was performed with Photoshop (function ‘photomerge'). Brightness and contrast was set again to optimize analysis conditions. The aligned images were measured in ImageJ. The unit of length was set on‘micrometre' and the pixel width on 0.168 under image properties. To measure the length of the several parts for IOD and fork speed, the selection tool and the function ‘measure' was used.

To get the track length in kbp, for the IODs, the value was multiplied with 2 (stretching-factor). For the fork speed in kbp min^−1^, the value was additionally divided by 30 (30 min nucleotide pulse).

### Dynamic cell cycle analysis

C2C12 stably expressing GFP-PCNA or HeLa Kyoto cells stably expressing FP-PCNA were plated on chambered glass coverslips one day before microscopy.

3D stacks were obtained on a UltraVIEW VoX spinning disc confocal system (Perkin Elmer, UK) in a closed live-cell microscopy chamber (ACU control, Olympus, Japan) heated to 37 °C, with 5% CO_2_ and 60% air humidity control, mounted on a Nikon Ti microscope (Nikon, Japan). Image acquisition was performed using a × 60/1.45 NA Planapochromat oil immersion objective lens. Images were obtained with a cooled 14-bit EMCCD camera (Hamamatsu) and had a voxel size of 104 × 104 × 500 nm^3^.

Alternatively, image time series were acquired with a Zeiss LSM 510 Meta laser scanning confocal microscope equipped with a stage mounted incubation system maintaining a humidified atmosphere of 5% CO_2_ at 37 °C (Okolab) using a 63 × /1.4 NA Plan-Apochromat oil immersion objective lens and the 488 nm laser line of an Argon ion laser at low power every 15 min (zoom=1.0, field size: 1,024 × 1,024 pixels; pixel size: 200 × 200 nm^2^) over 174 frames.

Individual frames were processed and assembled using ImageJ.

Visual inspection and classification of PCNA patterns frame by frame was performed and cells were first classified as: non-replicating, early/mid/late S-phase and mitotic. Temporal information on the preceding/subsequent cell cycle stage was used to discriminate between G1 and G2 cells.

The duration of each cell cycle sub-stage was determined by multiplying the number of frames corresponding to each cell cycle sub-stage by 15 min.

### Replication labelling and staining

For BrdU replication labelling, cells grown on cover glasses were incubated with 10–20 μM BrdU (BD Biosciences) for 5–30 min, fixed and stained as described above. Alternatively, cells grown on cover glasses were incubated with 10–20 μM EdU (Invitrogen) for the specified time, fixed and stained using the Click-iT assay (Invitrogen). Fluorophores conjugated to the secondary antibody or fluorescent azide were chosen to have sufficiently different emission spectra from the fluorescent group attached to FP-PCNA.

To enhance GFP-PCNA signal and increase signal-to-noise ratio before 3D-SIM imaging, C2C12 GFP-PCNA or HeLa Kyoto GFP-PCNA cells were processed as follows: cells were incubated with the CSK extraction buffer (10 mM Pipes-KOH, pH 7.0, 100 mM NaCl, 300 mM sucrose, 3 mM MgCl_2_) before fixing them as described in ref. 63. Fixed cells were permeabilized with 0.5% Triton X-100 and PCNA was detected using mouse anti-PCNA monoclonal antibody (1:200, Santa Cruz, catalog # sc-56) followed by goat anti-mouse IgG Alexa 488 (1:400, ThermoFisher Scientific, catalog # 11001). Stained samples were mounted in Vectashield (Invitrogen).

### Replication foci visualization and quantification

*Confocal microscopy*: Images were acquired with a Leica TCS SP5II confocal laser scanning microscope (Leica Microsystems, Wetzlar, Germany) equipped with an oil immersion Plan-Apochromat × 100/1.44 NA objective lens (pixel size in *XY* set to 50 nm, Z-step=290 nm) and laser lines at 405, 488, 561 and 633 nm. Alternatively, the spinning disk microscope was used (see dynamic cell cycle analysis section above).

*3D-SIM*: Super-resolution imaging of fixed samples was performed on a OMX prototype system^35^ or DeltaVision OMX V3 system (GE Healthcare) equipped with a × 100/1.40 NA PlanApo oil immersion objective (Olympus), Cascade II:512 EMCCD cameras (Photometrics) and 405, 488 and 593 nm diode lasers. Live-cell super-resolution imaging was performed with a DeltaVision OMX V3 Blaze system (GE Healthcare), equipped with a × 60/1.42 NA PlanApo oil objective and (Olympus) and sCMOS cameras (PCO) for high-speed stack acquisition. Both, fixed and live 3D-SIM was performed as previously described^64^.

3D-SIM super-resolution images were reconstructed^41^ by processing raw images using the API DeltaVision OMX softWoRx image processing software (version: 5.9.9 release 19).

For comparison, conventional wide-field image stacks were generated from 3D-SIM raw data by average projection of five consecutive phase-shifted images from each plane for the first rotation angle and subsequently subjected to an iterative 3D deconvolution using softWoRX 6.0. For direct comparison with 3D-SIM images, the pixel numbers were doubled in *x* and *y* using a bicubic interpolation in ImageJ to unify voxel sizes in all cases to 40 × 40 × 125 nm.

*Image analysis*: Quantification of RFi in cells was performed as summarized in Supplementary Fig. 3 and detailed in ref. 42. Briefly, confocal microscopy images were smoothed using mean filter (*r*=1.5) to reduce effects of noise on local maxima identification. Stacks were normalized and local maxima were identified and marked with single pixels having maximum intensity using ‘Find stack maxima' Image J macros available from: http://rsbweb.nih.gov/ij/macros/FindStackMaxima.txt. The stack with the map of local maxima was convolved with a Gaussian filter (*r*=1.0) to generate artificial focal objects around the identified maxima. Finally, the number of the objects corresponding to the local maxima was counted using a 3D object counting plug-in^65^ available from:

http://imagejdocu.tudor.lu/doku.php?id=plugin:analysis:3d_object_counter:start.

3D-SIM images were cropped with ImageJ to one nucleus only and background was removed automatically by the triangle method^66^. Volocity v.5 3D image analysis software (Perkin Elmer) was used to separate and count touching RFi (see Supplementary Fig. 3 for image preprocessing details).

### Statistical analysis representation

Statistical analyses were represented with violin plots (Supplementary Fig. 7; modified from ref. 29), a variation to the box plot with a kernel density plot on each side to display the distribution of the data at different values. Similar to a box plot it includes a marker for the median, a box indicating the inter-quartile range and whiskers for the upper and lower adjacent values.

### Error calculations

Error calculations were performed in R-Project (http://www.R-project.org).

S.d. for a single variable were computed via equation 1.


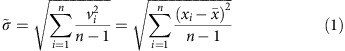


S.e.m. of the mean were calculated with equation 2.


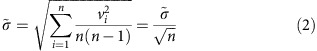


To calculate errors for diverse factors, for example, independent variables, the simplified version of the Gaussian error formula (the variance formula), as shown in equation 3, was used. Those errors were marked as ‘MSE'.


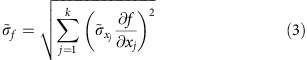


## Additional information

**How to cite this article:** Chagin, V. O. *et al*. 4D Visualization of replication foci in mammalian cells corresponding to individual replicons. *Nat. Commun.* 7:11231 doi: 10.1038/ncomms11231 (2016).

## Supplementary Material

Supplementary Information Supplementary Figures 1-7, Supplementary Table 1, Supplementary Notes 1-4 and Supplementary References

Supplementary Movie 1 Time-lapse confocal microscopy of dividing HeLa Kyoto cells expressing GFP-PCNA. Scale bar: 30 micron.

Supplementary Movie 2 Time-lapse 3D-SIM microscopy of replicating C2C12 cells expressing GFP-PCNA. Scale bar: 5 micron.

## Figures and Tables

**Table 1:** Cell cycle (stage) duration measurements.

**Cell cycle (stage)**	**HeLa Kyoto (h)**				**C2C12 (h)**			
	**Mean**	**s.d.**	**s.e.m.**	***n***	**Mean**	**s.d.**	**s.e.m.**	***n***
G1	8.9	1.8	0.3	31	8.8	3.4	0.7	20
S-phase	9.5	0.8	0.2	30	9.4	1.5	0.4	16
G2	3.7	0.7	0.1	27	3.7	0.8	0.3	5
Mitosis	0.9	0.2	0.0	26	0.8	0.2	0.1	10
Doubling time	22.6	2.3	0.4		22.6	2.5	1.0	

*n*, Number of cells.

**Table 2:** Genome size measurements.

**Cell line**	Relative DNA amount*	**Genomic DNA (pg)**	**Genome Size (10**^**3**^** Mbp)**	***n***
HeLa Kyoto GFP-PCNA	1.527	9.899	9.682±0.002	9
HeLa Kyoto mCherry-PCNA	1.544	10.007	9.786±0.006	8
C2C12 GFP-PCNA	1.798	11.676	11.419±0.006	4

^*^represents the ratio between the DNA amounts of the indicated cells in G1 and the DNA amount of G0/G1 C57Bl mouse splenocytes. All values are given as Mean±s.e.m.; *n* represents number of independent measurements.

**Table 3:** Statistics of the IOD measurements.

**IOD (kbp)**	**HeLa Kyoto**	**C2C12**
Mean	188.7	161.7
s.d.	121.4	100.3
s.e.m.	17.2	16.3
95% CI	33.6	31.9
*n*	50	38

CI, confidence interval; IOD, inter-origin distance; *n*, number of tracks.

**Table 4:** Statistics of the RFS measurements.

**RFS (10**^**3**^**Ntd per min)**	**HeLa Kyoto**	**C2C12**
Mean	1.65	2.46
s.d.	1.28	0.58
s.e.m.	0.12	0.11
95% CI	0.23	0.21
*n*	122	30

CI, confidence interval; *n*, number of tracks; RFS, replication fork speed.

**Table 5:** Calculation of the number of replicons per replication focus from the experimental data.

**Experimental data**	**Human HeLa Kyoto (Mean±s.e.m.)**	**Mouse C2C12 (Mean±s.e.m.)**
RFS, 10^3^ Ntd per min	1.65±0.12	2.46±0.11
IOD, kbp	188.7±17.2	161.7 ±16.3
GS, 10^3^ Mbp	9.7±0.002	11.4±0.006
Active RFi at any given time point	5,583±162	5,314±227
Total S-phase duration, minutes	570±9	564±23
**Calculations**	**Human HeLa Kyoto (Mean±MSE)**	**Mouse C2C12 (Mean±MSE)**
Time to replicate the genome with one fork (GS/RFS), hours	97,662±1.95 × 10^−5^	77,230±1.19*10^−5^
Replication forks active in parallel (GS/RFS/S-phase duration)	10,298±1	8,216±0.0
Replicons active in parallel (active forks/2)	5,149	4,108
Replicons per RFi (calculated replicons active in parallel/counted RFi)	**0.92**±**0.2**	**0.77**±**0.3**

GS, Genome size; IOD, Inter-origin distance; MSE, mean squared error; RFi, replication foci; RFS, replication fork speed.

See equation (3) in Materials and methods.
