# Supplementary material for: 4D Visualization of replication foci in mammalian cells corresponding to individual replicons
Source: Nat Commun. 2016 Apr 7;7:11231. doi: 10.1038/ncomms11231 (PMC4829660; doi:10.1038/ncomms11231)
Supplement: Supplementary Information — Supplementary Figures 1-7, Supplementary Table 1, Supplementary Notes 1-4 and Supplementary References [file ncomms11231-s1.pdf]

Supplementary Figure 1: Generation of HeLa Kyoto cell lines expressing PCNA tagged to fluorescent protein.

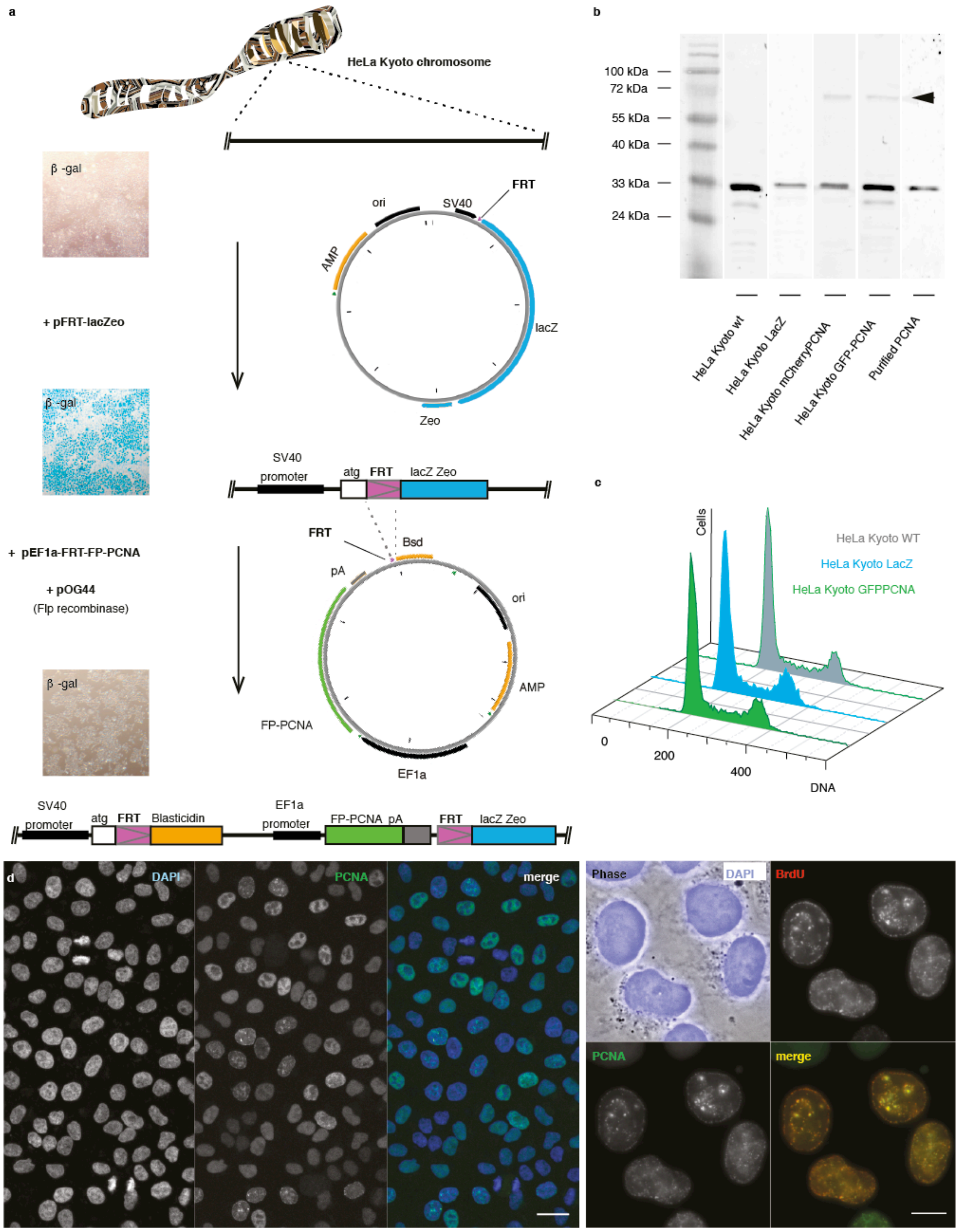

a. Schematic representation of the two-step protocol used to generate HeLa Kyoto cell lines stably expressing PCNA tagged to fluorescent protein – "FP-PCNA" (see Supplementary Note 1). HeLa Kyoto cell lines expressing mCherry-PCNA or GFP-PCNA proteins were generated by: First, introducing FRT site recognized by Flp recombinase together with LacZ and gene of Zeocin resistance. The cells where integration of the plasmid into a chromosome occurred were then selected on the basis of the acquired Zeocin resistance (eight days 75 µg/ml) and eight clones with integrated FRT sites were isolated. Beta-Galactosidase activity was assayed as an indicator of activity of SV40 promoter. Second, several HeLa-Kyoto-FRTLacZ-clones with low and high ß-galactosidase expression were selected for further co-transfection with FRT-GFP-PCNA or FRT-mCherry-PCNA containing plasmids with pOG44 plasmid containing gene of Flp-recombinase. Successful integration of FP-PCNA at the chromosomal FRT sites was verified based on acquired Blasticidin resistance and loss of LacZ activity. Scale bar: 50 micron. **b.** Immunoblot analysis of FP-PCNA expression levels in HeLa Kyoto cells at all stages of the protocol shown in (a). **c.** Flow cytometry histograms demonstrating the absence of alterations in the cell cycle dynamics. **d.** Left panel: microscopic images demonstrating the stability and uniformity of FP-PCNA expression. Scale bar: 10 micron. Right panel: colocalization of FP-PCNA with the nuclear sites of DNA synthesis. Scale bar: 5 micron.

Supplementary Figure 2: Criteria for inter-origin distance and replication fork speed sampling.

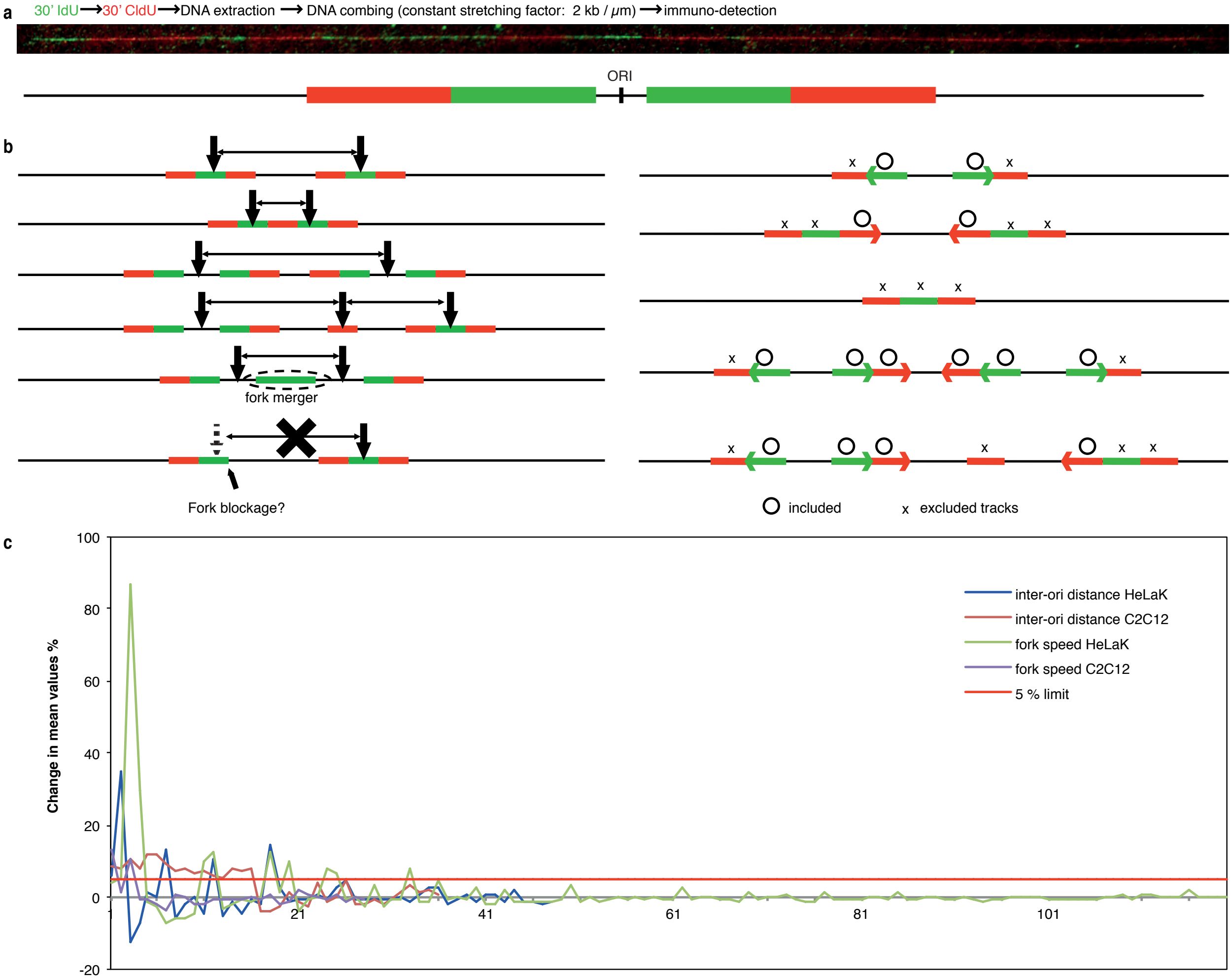

a. Schematic summary of the labeling, DNA fiber stretching and immunodetection steps. Exemplary image of stained DNA tracks acquired with a wide-field microscope. **b.** Fluorescent DNA fiber tracks were selected according to their pattern for calculations of inter-origin distances (IOD) and / or replication fork speed (RFS) as shown. Thick arrows indicate the positions considered as replication origins, thin arrows represent the IOD. Circles mark the tracks included into the calculations of RFS, crosses mark the tracks excluded from the calculations. **c.** The effect of increasing the sample size on the mean value of the indicated parameter is represented as a plot of the change in the average value of the sample "n" to the mean of "n-1" (See Supplementary Note 2).

Supplementary Figure 3: Protocols for *in situ* quantification of replication foci.

a

1) Segmentation of replication foci

Open nucleus image stack
    Remove slices w/o signal

Filter noise: Process>Filter>Mean;
    "Radius" = 1.5 pixels
    Process all slices
Normalize image stack: Process>Enhance contrast;
    Select: "Normalize",
    "Normalize all slices",
    "Use stack histogram",
    Enter: "saturated pixels" = 0.0%

Identify local maxima: Process>Find maxima;
    Select: "Preview point selection" and
    Choose noise tolerance setting above background signal
    Run "3D maxima" macros with the noise tolerance setting
    Select: "Output type" = "single points
    Save the output stack

2) Counting of replication foci

Counting of replication foci
Convolve the output stack with Gaussian: Process>Filter;
    Select: "Gaussian blur"
    "Radius" = 1.0 pixels
    Process all slices
    Normalize image stack

Count the local maxima: Plugins>"3D object counter"
    "Threshold" = 21 or 94

    "Maps to show" = "objects"
    "Results tables to show" = "Statistics", "Summary"

b

1) Segmentation of replication foci

Open nucleus image
Duplicate stack: image> Duplicate
    Select "Duplicate Stack"
    Select "OK"
With Duplicated image: image> Adjust> Autothreshold
    Choose "Triangle Method"
    Select "Ignore black"
    Select "White objects on black background"
    Select "Stack"
    Select "Use stack histogram"
    Select "OK"
Choose Process> image Calculator
    Image1: Choose thresholded image
    Specify method: "Min"
    Image2: Choose cropped original image
    Select "Create New Window"
    Select "OK"
Save resulting masked image

2) Counting of replication foci

Import masked image to Volocity
Select newly imported folder
Generate stack > Tools > Make volumes
Remove folder > Actions> Remove items
Choose newly generated volume
Set pixel sizes > Edit > Properties
Choose "Mesurements"
Drag task "Find objects using intensity" to measurement window
    Select wheel in top right corner to Specify intensities
    Choose "Lower" 1 & confirm With "OK"
Drag task "Separate touching objects" to measurement window

Step-by-step protocols for RFi identification and counting are summarized for: (**a**) confocal image stacks; (**b**) for 3D-SIM image stacks (See Supplementary Note 3).

Supplementary Figure 4: Replication foci clustering in late S-phase.

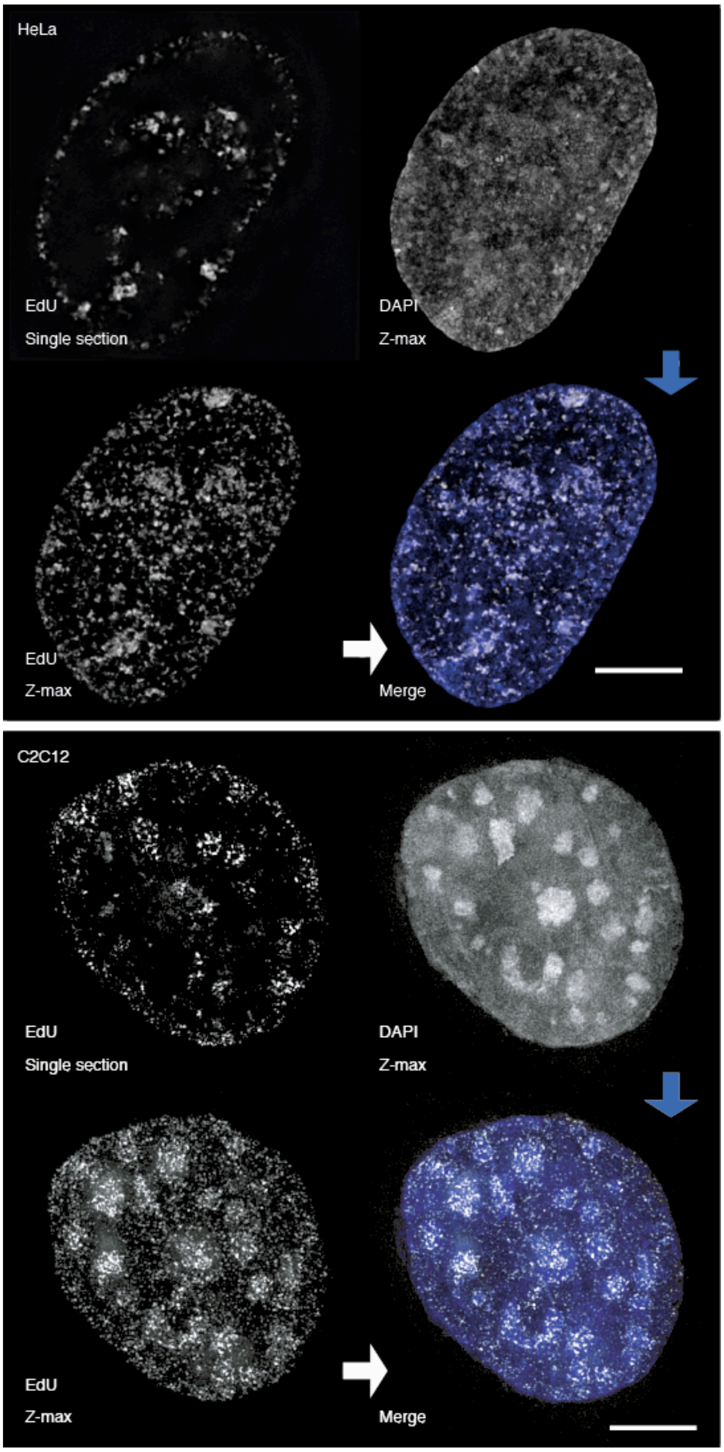

The degree of the observed heterochromatin associated clustering of EdU-labeled RFi in HeLa Kyoto cells (top panel). Replication foci labeled by demonstrate pronounced clustering during late S-phase in chromocenter regions of mouse C2C12 cells (bottom panel). See also Supplementary Note 4. Scale bar: 5 micron.

Supplementary Figure 5: Comparison of S-phase nucleotide (left) and protein (right) labeled replication foci numbers in HeLa Kyoto and C2C12 cells.

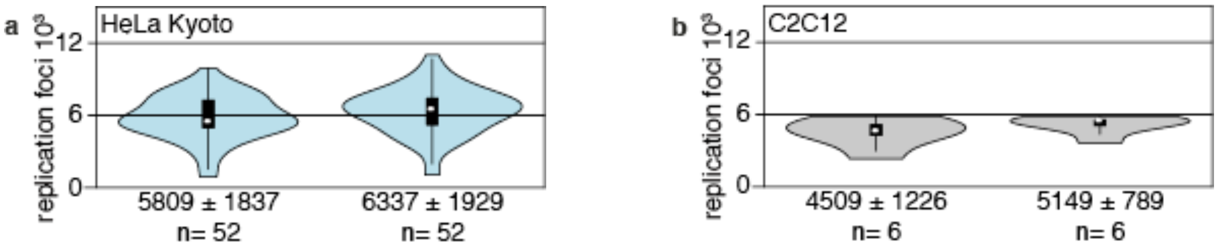

Details as in Figure 3. See also Supplementary Note 4.

Supplementary Figure 6: Schematic of different modes of replicon activation.

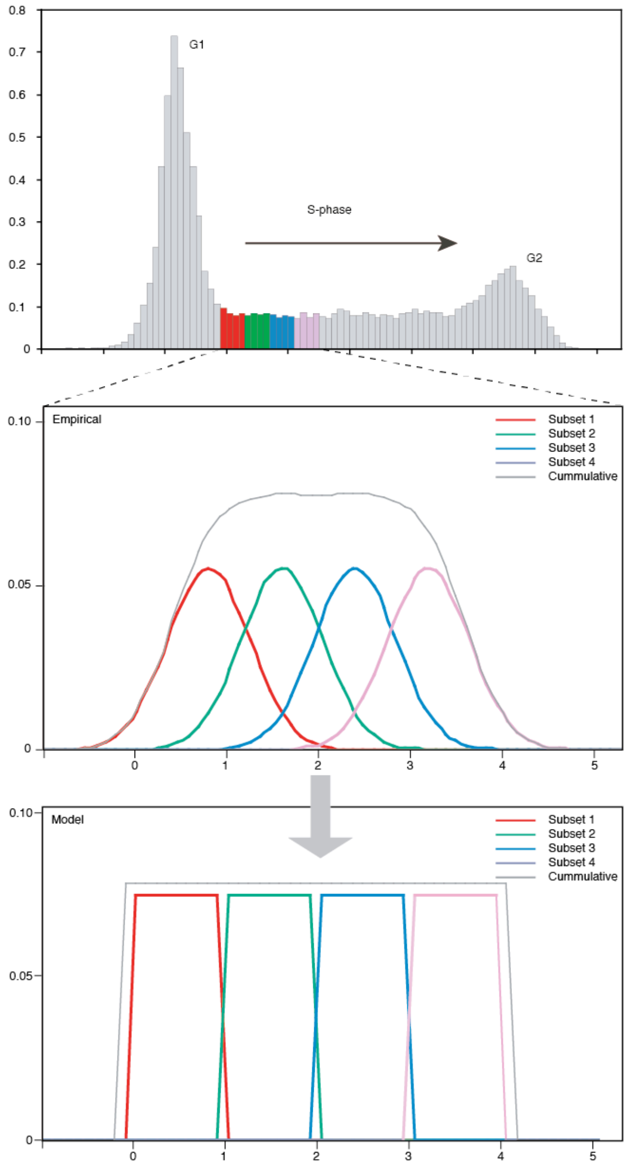

S-phase dynamics is represented as sequential activation of groups of replicons scattered over the genome. Each colored curve depicts combined intensity of DNA synthesis in a respective replicon subset. Cumulative DNA synthesis profile (S-phase part of DNA histogram, **top panel**) will be similar, if S-phase dynamics is modeled by asynchronous overlapping (Gaussian curves) mode of origin activation (**middle panel**), or as synchronized sequential (step-like curves) initiation of DNA synthesis (**bottom panel**) by the groups of replicons.

Supplementary Figure 7: Statistics representation using Violin plots.

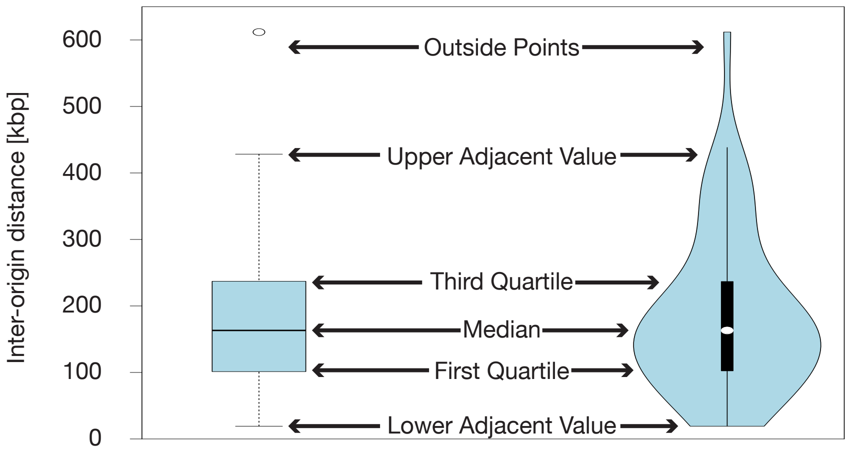

Supplementary Table 1: Summary of RFi measurements statistics.

S-phase stage	Cell line	Resolution	RFi mean	RFi median	SEM[1]	SD[2]	RFi min	RFi max	n[3]
early	HeLa Kyoto	confocal	1095.9	1072	22.4	157	839	1547	49
mid	HeLa Kyoto	confocal	1287.4	1301	23.9	141.6	941	1559	35
late	HeLa Kyoto	confocal	616.8	623	28.7	157.2	353	986	30
combined	HeLa Kyoto	confocal	1028.6	1081	28.2	300.9	353	1559	114
max	HeLa Kyoto	confocal	1175.7	1158	19.4	177.5	839	1559	84
early	C2C12	confocal	811	746	68	225.7	418	1162	11
mid	C2C12	confocal	1102	1072	63.1	282.3	517	1615	20
late	C2C12	confocal	818.5	857	46.8	203.9	366	1229	19
combined	C2C12	confocal	930.2	902	39.1	276.8	366	1615	50
max	C2C13	confocal	998.7	1035	53.1	295.7	418	1615	31
early	HeLa Kyoto	WFD	847.7	911	51.9	355.7	37	1476	47
mid	HeLa Kyoto	WFD	765	757.5	39.1	221	357	1269	32
late	HeLa Kyoto	WFD	503.8	488	34.6	265.6	16	1058	59
combined	HeLa Kyoto	WFD	681.5	703	28	328.8	16	1476	138
max	HeLa Kyoto	WFD	814.2	787	34.8	309.4	37	1476	79
early	C2C12	WFD	1011.5	1056	55.8	185	743	1398	11
mid	C2C12	WFD	847.1	937.5	53.4	213.5	437	1108	16
late	C2C12	WFD	642	689	63.5	210.7	49	840	11
combined	C2C12	WFD	835.3	848	39.8	245.2	49	1398	38
max	C2C13	WFD	914	964	41.4	215	437	1398	27
early	HeLa Kyoto	3D-SIM	5246	5703.5	227.6	2111	249	10976	86
mid	HeLa Kyoto	3D-SIM	6002.7	5699	218.9	1818.6	2326	11037	69
late	HeLa Kyoto	3D-SIM	3999.9	4059	291.6	2750.5	134	13178	89
combined	HeLa Kyoto	3D-SIM	5005.5	5206	155.5	2428.4	134	13178	244
max	HeLa Kyoto	3D-SIM	5582.8	5699	161.9	2015.5	249	11037	155
early	C2C12	3D-SIM	5462.4	5254	303.4	1689.2	2760	9279	31
mid	C2C12	3D-SIM	5236.9	4897.5	308.5	2389.5	1121	14394	60
late	C2C12	3D-SIM	3687.3	3987	220.1	1409.5	537	6436	41
combined	C2C12	3D-SIM	4808.5	4563	182.8	2100.1	537	14394	132
max	C2C13	3D-SIM	5313.7	5054	227.4	2169.3	1121	14394	91
combined	HeLa Kyoto	3D-SIM live	4216.7	3533	836.8	3131	652	11649	14
combined	C2C12	3D-SIM live	5026.8	5817.5	602.2	2408.9	61	8615	16

[1] standard error of mean

[2] standard deviation

[3] number of cells quantified

“max” values represent combined measurements from early and mid S-phases

Supplementary Note 1: Cell lines with stable expression of fluorescent DNA replication markers.

An important advantage of cell lines expressing fluorescent replication markers is the possibility of using them for live-cell analysis of the genome duplication process. Hence, in addition to our previously described mouse myoblast line expressing labeled GFP-tagged PCNA [1], we generated two human HeLa Kyoto cell lines variants expressing PCNA tagged to fluorescent proteins (FP-PCNA) by applying a two-step protocol of chromosomal integration based on Flp-mediated site-specific recombination (Supplementary Figure 1). By using different FP-PCNA constructs in the second step of the protocol, two cell lines were designed to express GFP-tagged PCNA and mCherry-tagged PCNA, respectively [1,2]. Both human cell lines revealed stable and uniform expression of fluorescently-tagged PCNA variants (Figure 1D, left panel) at about 12% of endogenous PCNA level, as measured by quantitative Western blot (Supplementary Figure 1B). We further verified by flow cytometry that the FP-PCNA proteins did not lead to cell cycle alterations (Supplementary Figure 1C). Similarly to native PCNA, recombinant FP-PCNA proteins localized in the nucleus and labeled sites of active DNA synthesis (Supplementary Figure 1D, right panel). Hence, we concluded that the generated HeLa Kyoto FP-PCNA cell lines exhibited unaltered replication dynamics [3,4] and represented an adequate model system for DNA replication in human cells.

Supplementary Note 2: Quantification of molecular replication parameters on combed DNA fibers.

The combination of double replication labeling and the fiber spreading protocol gave the possibility to discard fused or partially labeled replication fork tracks (Supplementary Figure 2) and provided increased precision for each measurement. At the same time, the number of tracks included into the quantifications was consequently reduced. Therefore, to ensure the relevance of our measurements we verified that the mean values of both measured parameters, RFS and IOD, were not affected by the sample size used. For that we varied the number of measurements used in the calculations and assessed the corresponding changes in the calculated mean values. For both parameters, individual measurements changed the mean values in less than 5% for the sample sizes used. Hence, we concluded that the number of replication fork tracks included in the calculations was sufficient for statistically significant measurements (Supplementary Figure 2C).

Supplementary Note 3: Development of computer-aided protocols for replication foci quantification.

Our initial tests revealed that very small threshold variations at the segmentation step could lead to substantial variations in the resulting RFi numbers. We therefore set out to develop approaches based on intrinsic image features that consequently would be as user-

independent as possible. Various algorithms for quantification of replication foci at the different resolution levels (confocal, wide field deconvolution and super-resolution 3D-SIM) were validated using parallel manual counting of RFi by independent persons. When assessing the outcome of RFi quantifications, preference was given to algorithms that resulted in fewer RFi (for a conservative estimate) than to algorithms that lead to overestimation of RFi numbers. The best correspondence between results of (semi)automatic RFi quantification and manual counting was obtained using the protocols described in Supplementary Figure 3 and in more detail in [5].

Supplementary Note 4: Influence of chromatin compaction and labeling mode on super-resolution imaging of replication foci.

Within highly compacted chromosomal regions perfect segmentation of foci even at super-resolution microscopy level is challenging (see Supplementary Figure 4). Hence, in particular in late S-phase mouse cells RFi numbers decreased. In addition, on average the number of RFi counted from PCNA labeling super-resolution images was slightly higher than the number of RFi labeled by nucleotide incorporation (Supplementary Figure 5). This difference could be due to an inefficient incorporation and/or detection of incorporated nucleotides. Alternatively, differences regarding dynamic behavior of DNA and PCNA components of RFi were revealed with super-resolution microscopy. It should be noted that during a 15-minute pulse of nucleotide incorporation an average replication fork labels about 25 kbp that correspond to eight microns of unpacked DNA. Therefore, up to certain condensation level the signal from the labeled nucleotide will be fuzzier than the focal signal from PCNA-containing replisomes. An underestimation of RFi numbers can be considered as a conservative estimate of actual RFi numbers and does not affect our further calculations and conclusions.

Supplementary References

1 Leonhardt, H. *et al.* Dynamics of DNA replication factories in living cells. *J Cell Biol* **149**, 271-280 (2000).

2 Rottach, A. *et al.* Generation and characterization of a rat monoclonal antibody specific for PCNA. *Hybridoma* **27**, 91-98 (2008).

3 Dimitrova, D. S. & Berezney, R. The spatio-temporal organization of DNA replication sites is identical in primary, immortalized and transformed mammalian cells. *J Cell Sci* **115**, 4037-4051 (2002).

4 Kennedy, B. K., Barbie, D. A., Classon, M., Dyson, N. & Harlow, E. Nuclear organization of DNA replication in primary mammalian cells. *Genes Dev* **14**, 2855-2868 (2000).

5 Chagin, V. O., Reinhart, M. & Cardoso, M. C. in *Methods Mol Biol* Vol. 1300 (eds J. Dalgaard & S. Vengrova) 43-65 (Springer Science+Business Media New York, 2015).
